# VirusFinder: Software for Efficient and Accurate Detection of Viruses and Their Integration Sites in Host Genomes through Next Generation Sequencing Data

**DOI:** 10.1371/journal.pone.0064465

**Published:** 2013-05-24

**Authors:** Qingguo Wang, Peilin Jia, Zhongming Zhao

**Affiliations:** 1 Department of Biomedical Informatics, Vanderbilt University School of Medicine, Nashville, Tennessee, United States of America; 2 Department of Psychiatry, Vanderbilt University School of Medicine, Nashville, Tennessee, United States of America; 3 Department of Cancer Biology, Vanderbilt University School of Medicine, Nashville, Tennessee, United States of America; 4 Center for Quantitative Sciences, Vanderbilt University School of Medicine, Nashville, Tennessee, United States of America; Wayne State University, United States of America

## Abstract

Next generation sequencing (NGS) technologies allow us to explore virus interactions with host genomes that lead to carcinogenesis or other diseases; however, this effort is largely hindered by the dearth of efficient computational tools. Here, we present a new tool, VirusFinder, for the identification of viruses and their integration sites in host genomes using NGS data, including whole transcriptome sequencing (RNA-Seq), whole genome sequencing (WGS), and targeted sequencing data. VirusFinder’s unique features include the characterization of insertion loci of virus of arbitrary type in the host genome and high accuracy and computational efficiency as a result of its well-designed pipeline. The source code as well as additional data of VirusFinder is publicly available at http://bioinfo.mc.vanderbilt.edu/VirusFinder/.

## Introduction

Viral infection, especially from tumorigenic viruses, is one of the leading causes of deaths worldwide. Some viruses, e.g. the hepatitis B virus (HBV), can fuse into a host genome ("integrated") to interrupt gene functions or induce chromosomal instability [1]–[4], while other viruses, e.g. the hepatitis C virus (HCV), rarely integrate into a host genome ("unintegrated"). Detecting the existence of viruses and, especially, their integration sites in host genomes is critical in understanding their molecular mechanisms in disease development.

With the rapid advances in next generation sequencing (NGS) technologies over the past several years and their increasingly widespread applications in clinical settings, recent large-scale investigations of virus-host interactions were carried out to shed light on virus-related cancers [2], [3], [5], [6]. The strong demand for the NGS investigation of virus-host interactions is currently hindered by the lack of effective NGS tools for virus detection. Recent tools such as PathSeq [7], RINS [8], and READSCAN [9] apply computational subtraction to pathogen detection in NGS data. However, these tools do not identify virus integration events, which are important for studying tumorigenic viruses like HBV in carcinogenesis [2], [3]. VirusSeq [10] uses whole transcriptome sequencing (RNA-Seq) data to detect virus integration sites in the human genome, yet is not practical for whole genome sequencing (WGS) data due to its high CPU requirements. Additionally, by including in its reference sequence 18 well-known viruses that have demonstrated potential to integrate into other genomes, VirusSeq is not able to identify integration sites of viruses other than the 18 predefined ones if without modification of its code. Another viral integration-detecting tool, ViralFusionSeq [11], can work with WGS data in addition to RNA-Seq, but is not applicable to samples infected with undiagnosed viruses; rather, it requires virus sequence in advance as a pre-defined input. All these tools were recently developed, and their computational capacities and accuracy remain largely uncertain.

To address the above limitations, here, we introduce VirusFinder for efficient and accurate identification of viruses and their integration sites (if present) from NGS data. Specifically, VirusFinder aims to: (i) detect the presence of viruses of arbitrary types in a host sample, whether they are integrated or unintegrated; (ii) detect virus insertion sites as long as the virus fuses into the host genome and its integration sites are captured by sequencing technologies; and (iii) work on WGS, RNA-Seq or targeted sequencing data. VirusFinder does not require virus sequences as a prerequisite input. Hence, it can not only work with NGS data with a specified virus type, like VirusSeq and ViralFusionSeq, but also be applied readily to samples infected with undiagnosed viruses, to which neither VirusSeq nor ViralFusionSeq is directly applicable.

## Methods

**Figure 1:**
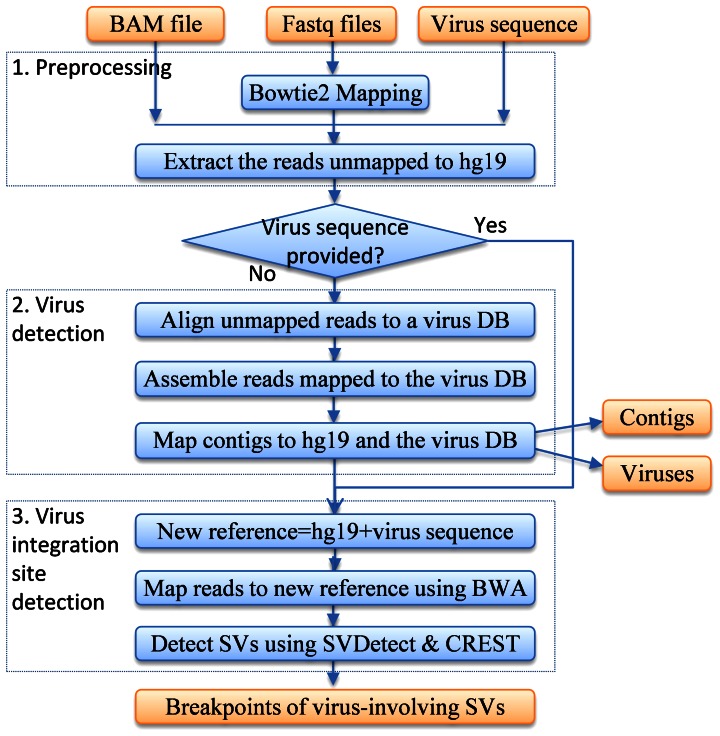
VirusFinder’s pipeline to detect viruses and their integration sites in next generation sequencing (NGS) data. VirusFinder overall follows a three-step procedure: (1) preprocessing, (2) virus detection, and (3) virus integration site detection. The current version of VirusFinder (release 1.0) uses UCSC hg19 (http://hgdownload.cse.ucsc.edu/downloads.html#human) as reference human genome. DB: database. SVs: structural variants (especially inter-chromosomal translocations).

Figure 1 illustrates the pipeline of VirusFinder, which overall follows a three-step procedure: (1) preprocessing, (2) virus detection, and (3) virus integration site detection. These steps are elaborated in detail in the text below.

### (1) Preprocessing

VirusFinder’s input can either be raw sequencing reads (in Fastq format) or an alignment file (in BAM format). If user provides only raw sequencing reads, VirusFinder will first use the alignment tool Bowtie 2 [12] to map these reads to a human reference genome, which can either be NCBI build 37/36 (http://www.ncbi.nlm.nih.gov/projects/mapview/) or UCSC hg19/hg18 (http://hgdownload.cse.ucsc.edu/downloads.html#human). VirusFinder runs Bowtie 2 in its sensitive end-to-end mode, in which Bowtie 2 does not trim (or "soft clip") characters from short reads in order to achieve high alignment speed. With the alignment file generated by Bowtie 2 or provided by the user, VirusFinder then garners all reads unmapped to the human reference genome for downstream analysis.

Here in this step, user is allowed to provide the sequence of the virus being examined as an input parameter of VirusFinder. VirusFinder will skip step (2) of the pipeline if user provides the virus sequence.

### (2) Virus detection

This step is used to detect the specific type(s) of virus(es) present in the sample. This step will be skipped if user supplies the virus sequence to VirusFinder. If the virus type is unknown, however, VirusFinder first aligns the unmapped reads collected in step (1) to a virus database (virus DB). The current version of VirusFinder (release 1.0) uses the same virus DB, virus.fa, as the one included with the RINS package (http://khavarilab.stanford.edu/resources.html) [8]. This virus DB contains viruses of all known classes (32,102 in total) [8]. User can replace virus.fa with an alternative virus DB, Genome Information Broker for Viruses (GIB-V) [13] (http://gib-v.genes.nig.ac.jp/), which collects 25,525 virus reference sequences, or a smaller set of viruses of user interest.

Next, VirusFinder *de novo* assembles the reads aligned to the virus DB into contigs and maps contigs to both the human genome and the virus DB. All contigs that are mapped to the human genome are discarded. The alignment scores of the nonhuman contigs, which align only to the virus DB, are then used to rank the viruses, to which they are mapped. The sequence of the top ranking virus is then applied to the next analysis step.

It may be worth mentioning that our virus detection method as described here used RINS [8] as a starting point. However, different from RINS that identifies viruses by recruiting all reads mapped to the virus DB, which can at the same time align to the human genome, VirusFinder utilizes only the reads mapped to the virus DB and unmapped to the human genome for virus detection. By using less reads than RINS and more importantly with a simplified pipeline, VirusFinder achieves significant speedup over RINS without sacrificing its accuracy. We have tested VirusFinder on more than 20 samples (including unpublished ones) infected with viruses of various types and VirusFinder detected correct virus types for all the test samples (see section below for results on publically available data).

### (3) Virus integration site detection

VirusFinder combines the human reference genome with the virus sequence (designated as a separate pseudo-chromosome, chrVirus) identified in previous step (2) or provided by the user. It then uses the mapping tool BWA [14] to align the reads recruited in step (1) to this new reference. Another tool VirusSeq [10] also concatenates the human genome with virus sequences. But VirusSeq includes a fixed set of, i.e. 18, virus sequences in its reference genome and hence cannot be applied directly to detect virus insertion sites in samples infected with viruses other than the18 predefined ones. By concatenating the viruses detected in step (2) on the fly, VirusFinder is readily applicable to samples harboring viruses of arbitrary types (as long as they are represented in the virus DB).

From the resultant alignment file, VirusFinder calls inter-chromosomal structural variants (SVs) using CREST [15]. The breakpoints of the SVs that involve both the virus and human genome, if there are any, are then reported. CREST utilizes soft-clipped reads as breakpoint positions of SVs. On a WGS sample with a modest 30× coverage, CREST can take several days to complete. To speed up our pipeline, before executing CREST, we run a much faster SV-detecting tool SVDetect [16] on the alignment file to calculate potential regions harboring virus integration sites. Then we modified CREST to make it search primarily within the regions identified by SVDetect. By blending SVDetect with CREST, we are able to reduce the computational time of SV calling significantly from several days on a WGS sample to around an hour.

When the pipeline terminates, three files, ‘virus.txt’, ‘contig.txt’ and ‘integration-sites.txt’, are created in the working directory of VirusFinder. These files contain candidate viruses identified by VirusFinder, contigs mapped to these virus sequences, and detected virus insertion sites, respectively. For each virus insertion event, VirusFinder provides its breakpoints in both the virus sequence and the human genome. For detailed explanations of these files, please read our user manual in the Supplementary Material S1.

### (4) Software implementation

The entire pipeline of VirusFinder, from the initial preprocessing step to the final virus integration site detection, is fully automated. As far as we know, this is the first fully automatic pipeline combining virus detection (step 2) seamlessly with virus integration site identification (step 3) and, thus, the first NGS software enabling the automatic detection of virus integration sites in samples for which viruses may not necessarily be determined beforehand. The aforementioned tool VirusSeq provides both virus detection script and virus integration site identification script too. Unfortunately, in VirusSeq, they are separate programs and cannot work directly together. Another advantage of VirusFinder is that it is capable of analyzing large-scale NGS data efficiently by improving significantly its computational pipelines for viruses and their integration sites detection. VirusFinder further improves its analysis speed by blending a fast aligner Bowtie 2 in the time-consuming step (1) with a slower yet more sensitive aligner BWA on a smaller subset of reads in step (3).

VirusFinder was implemented in Perl programming language and has been tested on various Linux platforms. It depends on several third-party tools, including BLAST+ (or BLAST) [17], BLAT [18], SAMtools [19], and Trinity [20], in addition to the aforementioned Bowtie 2, BWA, SVDetect, and CREST. All these tools are publically available. Their download URLs and brief descriptions are provided in the Supplementary Material S1. Different from other tools, CREST requires the installation of a BLAT server. To ease the distribution of VirusFinder, we modified CREST into a standalone tool, which, together with several other software that CREST requires, is now included in the release package of VirusFinder. This removed completely the requirement to install a BLAT server on user’s system.

## Results

To evaluate VirusFinder, we compared VirusFinder with the aforementioned VirusSeq [10] and ViralFusionSeq [11], two publically available tools emerged recently for characterizing virus integration sites in host genomes.

### Virus detection

We firstly tested the ability of VirusFinder to detect the presence of viruses in human samples. Table 1 lists the samples we collected for our benchmark experiment. These samples were infected with viruses of diverse types and six of them were confirmed to harbor virus integration sites. The first two samples in Table 1 were generated using WGS [2], the next two using RNA-Seq [7], [11], and the remaining three targeted sequencing [21], [22]. The first three samples are the complete test data set of ViralFusionSeq and the fourth one, HeLa cervical cancer cell line, was used to evaluate PathSeq [7].

**Table 1:** Detection of viruses in seven NGS samples using VirusFinder and VirusSeq.

Sample	Sequencing technology	Virus	#Virus integration sites	VirusFinder	VirusSeq
HCC sample 198T	WGS	HBV	2	√	–
HCC sample 268T	WGS	HBV	3	√	–
HCC cell line HKCI-5a	RNA-Seq	HBV	3	√	×
HeLa cervical cancer cell line	RNA-Seq	HPV-18	1	√	×
MCC case 27	Targeted sequencing	MCV	1	√	√
MCC case 36	Targeted sequencing	MCV	2	√	√
An Indian patient with fever and acute encephalitis	Targeted sequencing	JEV	0	√	√

HCC: Hepatocellular carcinoma. MCC: Merkel cell carcinoma. WGS: whole genome sequencing. RNA-Seq: whole transciptome sequencing. √: detected. ×: failed. –: software did not end within allowable time.

As indicated in Table 1, VirusFinder identified the correct virus types in all the test samples. We also evaluated VirusFinder using additional 8 WGS samples (see the Computational efficiency section below) of different sequencing coverage, including 5 Hepatocellular carcinomas (HCCs) and 3 normal tissues. VirusFinder identified correct virus types for all these WGS samples and hence demonstrated its robustness in virus detection.

Table 1 shows that VirusSeq reported a false virus type for HCC cell line HKCI-5a. It also failed to detect the presence of HPV-18 virus in the HeLa cervical cancer cell line (VirusSeq failed probably because MosaikAligner, the mapping tool used in VirusSeq, extracted zero unaligned reads from the sequencing data of this cell line). ViralFusionSeq does not detect virus type and hence was not touched on here.

### Virus integration site detection

We utilized the samples in Table 1 that harbor virus integration sites as test data to evaluate the virus insertion loci predicted by VirusFinder. Here, we excluded the HeLa cervical cancer cell line from this experiment, because a large chromosomal region in 8q24 instead of a precise virus insertion site was reported for this sample [7].

Table 2 presents the virus insertion loci detected by the three methods. The real virus insertion positions are in Column 2. From Table 2, we can see that on the two WGS samples, 198T and 268T, both VirusFinder and ViralFusionSeq pinpointed all the exact integration breakpoints reported in a recent study by Sung *et al.* [2]. On the two targeted sequencing samples, MCC cases 27 and 36, although the three integration positions predicted by VirusFinder are not as accurate as those on the WGS samples, they are comparable to or slightly more accurate than those detected by VirusSeq.

**Table 2:** Detection of virus integration sites in five NGS samples^a^.

Sample	Integration sites	VirusFinder	VirusSeq	ViralFusionSeq
HCC sample 198T	chr5:1,269,387	chr5:1,269,387	–	chr5:1,269,387
	chr5:1,269,405	chr5:1,269,405		chr5:1,269,405
HCC sample 268T	chr5:1,292,391	chr5:1,292,391	–	chr5:1,292,391
	chr5:1,292,403	chr5:1,292,403		chr5:1,292,403
	chr19:30,298,787	chr19:30,298,787		chr19:30,298,787
HCC cell line HKCI-5^b^	N/A	chr7:98,532,319	chr7:98,532,182	∼chr7: 98,532,184- 98,532,285
	N/A	chr16:30,407,194	chr16:30,408,118	∼chr16:30,408,324
MCC case 27	chr9:121,417,276	chr9:121,417,092	chr9:121,417,087	×
MCC case 36	chr13:99,978,184	chr13:99,978,244	chr13:99,977,889	×
	chr13:97,820,362	chr13:97,820,192	chr13:97,820,189	

N/A: not available. –: software did not end within allowable time. ×: software failure.^ a^Only the samples in Table 1 that harbor virus integration sites are included in this table (HeLa cervical cancer cell line was excluded from this table because a large chromosomal region in 8q24 instead of a precise virus insertion position was provided for this sample [7]). ^b^It is the test data of ViralFusionSeq [11]. The virus integration sites of this sample were validated but are not publically available. ViralFusionSeq outputs human-virus fusion sequences instead of fusion breakpoints. Its predictions of virus integration sites for the first two samples, 198T and 268T, were taken from its published paper. When running ViralFusionSeq on the sample HCC cell line HKCI-5, we got the intermediate results that indicate a virus integration site around chr16:30408324. Its user manual provides another position, chr7: 98532184- 98532285, for this sample. Both loci were included here for the purpose of comparison.

### Computational efficiency

Finally, we compared the computation time of the 3 tools on 10 WGS samples infected with HBV virus (Table 3). All these samples are from the study by Sung *et al.* [2]. In their original study, Sung *et al.* validated 22 out of 176 WGS samples. Limited by computational resources, we picked 10 out of the 22 validated samples so that: (i) the hotspots of the integration breakpoints in both the human genome, e.g. genes *TERT*, *MLL4* and *CCNE1*, and the HBV virus genome, e.g. locus 1800, are covered; (ii) our test data includes not only tumor samples but also normal tissues – 200N, 268N, and 180N are three normals; (iii) different sequencing depths, which range from 31.7× to 121.2×, are represented; (iv) our test data includes the aforementioned two samples, 198T and 268T, so as to use them to compare VirusFinder with ViralFusionSeq (they were the test data of ViralFusionSeq).

**Table 3:** Comparison of computation time of three virus integration-detecting methods on whole genome sequencing (WGS) data^a^.

Sample	Coverage	VirusFinder		ViralFusionSeq^b^		VirusSeq^c^	
		#CPUs	Time (days)	#CPUs	Time (days)	#CPUs	Time (days)
26T	65.5×	8	3.1	8	17.8	8	>7
71T	32.2×	8	1.9	8	11.5	8	>7
106T	44.8×	8	2.4	8	17.1	8	>12.5
180N	121.2×	8	7.3	8	>17.4	8	>12.5
186T	36.5×	8	2.0	8	13.0	8	>12.5
198T	34.4×	8	1.8	8	10.8	8	>12.5
200N	32.6×	8	1.9	8	11.5	8	>12.5
200T	31.7×	8	2.0	8	12.5	8	>12.5
268N	40.7×	8	2.7	8	14.5	8	>12.5
268T	34.1×	8	2.0	8	13.5	8	>9.9
Average			2.7		14.0		>11.1

a The computation time of the three methods on these samples were analyzed on Vanderbilt Advanced Computing Center for Research & Education (ACCRE, http://www.accre.vanderbilt.edu/), with the same configuration of CPUs in each node.

b ViralFusionSeq did not terminate successfully on sample 180N.

c We attempted to run VirusSeq three times on these WGS samples. The first trial failed because the size of its intermediate files exceeded our cluster quota. After getting more space, we reran VirusSeq. After non-stop running for a whole week, all our jobs were killed in server due to their exceeding allocated time – not realizing initially the long computation time of VirusSeq on WGS samples. In our latest trial of VirusSeq on February 13, 2013, we requested 35 GB memory, 8 CPUs, 30 days for each job and resubmitted our jobs to ACCRE. Seven jobs were scheduled to run first. After twelve and a half day, all these jobs were killed due to an unexpected internal network outage of ACCRE. Though we were not able to make VirusSeq terminate successfully on these WGS samples due to expensive computing, we may conclude from the data that VirusFinder runs much faster than VirusSeq.

The computation time of the three tools on these samples were analyzed on Vanderbilt Advanced Computing Center for Research & Education (ACCRE, http://www.accre.vanderbilt.edu/), with the same configuration of CPUs in each node. The memory we requested for each job of VirusFinder, ViralFusionSeq, and VirusSeq was 20 GB, 40 GB and 35 GB, respectively.

The average running time of these three tools is shown in Table 3. Based on 8 processors, VirusFinder and ViralFusionSeq took on average 2.7 and 14.0 days, respectively, on a WGS sample, while VirusSeq did not terminate within the allowable time (>11.1 days) on all the test samples. Among the 10 samples, 180N has the highest coverage (121.2×) and is the only normal sample validated to harbor a HBV fusion site [2]. Among the three methods, only VirusFinder successfully detected the HBV virus and its integration sites in 180N within the allocated time. This indicates that VirusFinder is capable of analyzing WGS samples with very high sequencing coverage. Both ViralFusionSeq and VirusSeq failed on this sample primarily due to their exceedingly high CPU requirements.

## Discussion

With the increasing interest in applying NGS to investigate virus-host interactions in human cancer, new software tools emerged recently to detect viruses and their integration sites in the human genome. Unfortunately, these tools could hardly meet the challenges posed by the rapidly advancing NGS technologies of today, due to their limited capability and low computational efficiency. This is the main reason why we developed VirusFinder as introduced in this paper.

To the best of our knowledge, VirusFinder is the first fully automatic pipeline capable of characterizing integration loci of undiagnosed viruses of arbitrary types in NGS data. The results of our evaluation indicated that VirusFinder can accurately detect viruses and their integration sites in the human genome. The benchmark experiment on 10 WGS samples also demonstrated that VirusFinder is ideal for quick and accurate analysis of large-scale NGS data. These we believe will greatly benefit the studies that utilize NGS to investigate the etiologic association of viruses with disease, especially human cancer.

## Supporting Information

Supplementary Material S1 **VirusFinder’s user manual.**
(PDF)
