# Supplementary material for: VirusFinder: Software for Efficient and Accurate Detection of Viruses and Their Integration Sites in Host Genomes through Next Generation Sequencing Data
Source: PLoS One. 2013 May 24;8(5):e64465. doi: 10.1371/journal.pone.0064465 (PMC3663743; doi:10.1371/journal.pone.0064465)
Supplement: Supplementary Material S1 — VirusFinder’s user manual. (PDF) [file pone.0064465.s001.pdf]

VirusFinder (version 1.0)

USER'S MANUAL

VANDERBILT

Bioinformatics and Systems Medicine Laboratory
Vanderbilt University Medical Center
Nashville, Tennessee, USA

March, 2013

TABLE OF CONTENTS

Page No.

1. Introduction 1

2. Installation 1

2.1 Source code 1

2.2 Third-party tools 2

2.3 Reference genome 2

3. Software Input 3

3.1 Input parameters of VirusFinder.pl 3

3.2 Configuration file 3

4. Software Output 5

4.1 Intermediate results 5

4.2 Output of virus detection 5

4.3 Output of integration site detection 6

5. Reference *7*

1. INTRODUCTION

VirusFinder is a software tool for efficient and accurate identification of viruses and their integration sites (if present) in host genomes (e.g. the human genome) from next generation sequencing (NGS) data, including whole genome sequencing (WGS), whole transcriptome sequencing (RNA-Seq), or targeted sequencing data. VirusFinder follows a three-step procedure: (1) preprocessing, (2) virus detection, and (3) virus integration site detection. Step (2) can be skipped if the sequence of the virus being examined is provided as an input parameter of VirusFinder. If the virus type is unknown, however, VirusFinder will run all the three steps sequentially on the input data. Figure 1 shows the flowchart of virus identification using VirusFinder.

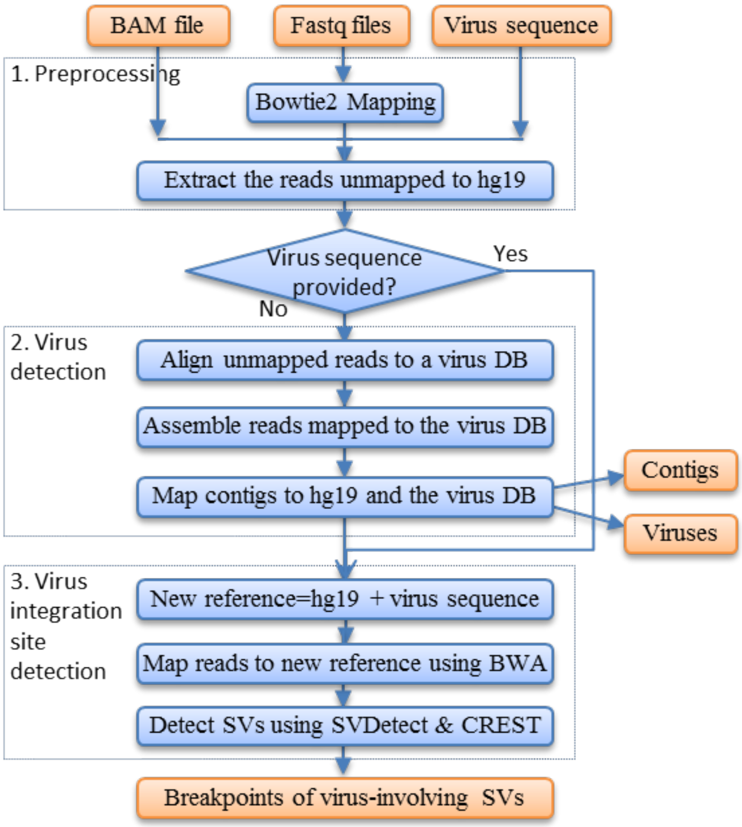

Figure 1: Flowchart of VirusFinder. DB: database. SVs: structural variants.

2. INSTALLATION

2.1 Source code

We used Perl to implement VirusFinder. The following table provides a summary of all our Perl scripts, among which VirusFinder.pl is the interface of VirusFinder. VirusFinder.pl prepares input data for each of its three steps and processes their output after they terminate. Scripts detect_virus.pl and detect_integration.pl correspond to the steps (2) and (3) of the pipeline, respectively. They have independent functions and, hence, can also be run separately. For the latest version of VirusFinder, please check our website http://bioinfo.mc.vanderbilt.edu/VirusFinder/.

Table 1: Source code of VirusFinder

Script	Description
VirusFinder.pl	The interface of VirusFinder. It runs other Perl scripts to do actual work.
preprocess.pl	Preparing input data for scripts, detect_virus.pl and detect_integration.pl.
detect_virus.pl	Detecting the existence of viruses in the data. If a virus sequence is provided to VirusFinder.pl, this script will be skipped by VirusFinder.pl.
detect_integration.pl	Detecting the integration sites of viruses in host genomes.
Mosaic.pm	A Perl module consisting of subroutines shared by other Perl scripts.
sys_check.pl	An auxiliary script to check if the required CPAN modules are installed.

2.2 Third-party tools

Table 2 lists third-party tools required by VirusFinder. Users should specify the full paths to these tools in a configuration file (see subsection 3.2 for a detailed description of the configuration file).

Table 2: Third party tools used in VirusFinder

Tool	Version	URL	Description
BLAST+	2.2.26+	ftp://ftp.ncbi.nlm.nih.gov/blast/executables/blast+/	Alignment tool
BLAT	V.34	http://genome.ucsc.edu/cgi-bin/hgBlat	Alignment tool
Bowtie2	2	http://bowtie-bio.sourceforge.net/bowtie2/	Alignment tool
BWA	0.6.1	http://bio-bwa.sourceforge.net/	Alignment tool
SAMtools	0.1.18	http://samtools.sourceforge.net/	Alignment processing tool
Trinity	2012-06-08	http://trinityrnaseq.sourceforge.net/	De novo assembly tool
CREST[1]	1.1	http://www.stjuderesearch.org/site/lab/zhang	Structural variant calling
SVDetect	r0.8	http://svdetect.sourceforge.net/Site/	Structural variant calling

[1]An in-house version of CREST as well as several other tools it requires is provided in our release package under the directory *bin*. Hence, there is no need for user to install CREST or specify its path in the configuration file.

VirusFinder also needs several Perl modules, e.g. threads.pm, to make it work properly. The script sys_check.pl in Table 1 can help you identify modules that need to be installed in your system.

2.3 Reference genome

2.3.1 Human reference genome

Human reference genome in FASTA format is required for VirusFinder. Currently, the reference genome used in VirusFinder is UCSC hg19 (http://hgdownload.cse.ucsc.edu/downloads.html#human). The FASTA file hg19.fa needs to be indexed. The Bowtie2, BWA and BLAST+ indices can be created using the following command lines.

```
bowtie2-build hg19.fa  hg19
bwa index hg19.fa
makeblastdb –in hg19.fa –dbtype nucl –out hg19
```

2.3.2 Virus database (DB)

We used the same virus database (DB), virus.fa, as the one included with the RINS package (Bhaduri *et al.*, 2012, http://khavarilab.stanford.edu/resources.html). This virus DB contains viruses of all known

classes (32,102 in total) (Bhaduri *et al.*, 2012). User can replace virus.fa with an alternative virus database, Genome Information Broker for Viruses (GIB-V, http://gib-v.genes.nig.ac.jp/). VirusFinder requires the BLAST+ index of the virus DB, which can be created using the following command:

makeblastdb -in virus.fa -dbtype nucl -out virus

The full directories of the human reference genome and virus DB should be specified in the configuration file (see subsection 3.2).

3. SOFTWARE INPUT

3.1 Input parameters of VirusFinder.pl

VirusFinder.pl prints the following help information (Figure 2) if it is run with no argument or with the argument "-h".

```
Please provide a configuration file!

Program: VirusFinder, a tool for identifying virus sequences and their integration sites.
Version: 1.0 (2/1/2013)

Usage: VirusFinder.pl -c <configuration file> [options]

Options:
  -h, --help      Displays this information
  -c, --config    Configuration file <required>
  -v, --virus     The sequence of the virus present in the sample, not required
  -o, --output    The directory to store software output, default is current working directory
```

Figure 2: Help information of VirusFinder.

The help information indicates that a configuration file is required by VirusFinder as a mandatory input. We will use entire subsection 3.2 below to discuss the configuration file due to its importance.

Besides the configuration file, user can specify/provide the sequence of the virus under study (in FASTA format) to VirusFinder, if the virus that infected the sample is already known. As VirusFinder has the capability to determine the correct type of the virus in the sample, the option "-v" is not required. However, because virus detection is time-consuming, the offer of virus sequence to VirusFinder can bring significant timesaving.

All the intermediate and final results of VirusFinder are stored in the directory specified by the option '-o'. If the output directory is not provided, current working directory will be used.

3.2 Configuration file

The configuration file allows users to specify the full paths to their sequencing data and the third-party tools required by VirusFinder. An example configuration file is provided in Figure 3, which shows different input parameters are defined as "variable=value" pairs.

```
##################################
## Input NGS data can be either of the following two options:
##  (a) an alignment file (in BAM format),
##  (b) two paired-end FASTQ files.
##################################

alignment_file  = /scratch/kingw/virusFinder/simulation/simulation..bam
#fastq1         = /scratch/kingw/virusFinder/simulation/seq_1.fastq.gz
#fastq2         = /scratch/kingw/virusFinder/simulation/seq_2.fastq.gz

mailto          = qingguo.wang@vanderbilt.edu
thread_no       = 8     #the number of threads for parallel computing


##################################
## The full paths to the following third-party tools are required by VirusFinder:
##################################

samtools_bin     = /scratch/kingw/bin/samtools-0.1.18/samtools
blat_bin         = /scratch/kingw/bin/blat
blastn_bin       = /scratch/kingw/bin/ncbi-blast-2.2.26+/bin/blastn
bowtie_bin       = /scratch/kingw/bin/bowtie2-2.0.2/bowtie2
bwa_bin          = /scratch/kingw/bin/bwa-0.6.1/bwa
trinity_script   = /scratch/kingw/bin/trinityrnaseq_r2012-06-08/Trinity.pl
SVDetect_dir     = /scratch/kingw/bin/SVDetect_r0.8


##################################
## Reference files (indexed for Bowtie2, BWA, and BLAST)
##################################

virus_database         = /scratch/kingw/virusFinder/ref/virus.fa
bowtie_index_human = /scratch/kingw/virusFinder/ref/hg19
blastn_index_human  = /scratch/kingw/virusFinder/ref/hg19
blastn_index_virus    = /scratch/kingw/virusFinder/ref/virus


##################################
## Modifiable parameters (users are suggested not to modify them)
##################################

min_contig_length  = 300
blastn_evalue_thrd  = 0.05
similarity_thrd        = 0.8
chop_read_length    = 25
minIdentity            = 80
```

Figure 3: An example configuration file.

The first three variables in the above configuration file specify the paths to the NGS data to be analyzed. The input of VirusFinder can either be raw sequencing reads (in FASTQ format) or an alignment file (in BAM format). All the rest of the variables except "mailto" are mandatory.

4. OUTPUT

Under the working directory of VirusFinder, three files, 'virus.txt', 'contig.txt' and 'integration-sites.txt', which contain the final results, will be created upon the termination of the method. These files as well as the intermediate files generated by VirusFinder are introduced in subsections below.

4.1 Intermediate results

VirusFinder keeps all intermediate results so that it does not have to restart the whole process from scratch if the server, to which it is assigned to run, fails. The drawback of this design is that users may have to manually delete the file that is improperly created by VirusFinder as a result of system failure.

Typically, each run of VirusFinder creates three folders, 'step1', 'step2', and 'step3', under the directory specified by users. Each of these three folders stores the intermediate files of the corresponding step of VirusFinder. For WGS data with very high coverage, e.g., 120×, the size of the intermediate files in the folder 'step1' can be close to 0.5TB. So we remind users to delete them after the analysis process terminates.

4.2 Output of virus detection

At the end of step (2), i.e., the virus detection step, two resultant files are copied out of the subdirectory 'step2' to be placed in the working directory of VirusFinder. These two tab-delimited files, named 'virus.txt' and 'contig.txt' respectively, contain candidate viruses identified by VirusFinder and the corresponding contigs mapped to the virus sequences.

The candidate viruses in the file 'virus.txt' are sorted based on the alignment quality of the contigs that are mapped to them. The top-ranking virus candidate is in the first row of this file. Table 3 below shows the top three candidate virus sequences identified from our simulation data, which can be downloaded from our website (http://bioinfo.mc.vanderbilt.edu/VirusFinder/). All these three candidates indicate the existence of HPV-16 virus in the sample and HPV-16 is exactly what we inserted into this simulation data initially. One contig, comp4_c0_seq1, map to all these three candidate sequences. The length of the contig and the number of reads fallen on it are also provided in the file 'virus.txt'.

It is worth mentioning that at the end of step (2), VirusFinder.pl extracts the sequence of the top-ranking virus, i.e. 'gi_310698439_ref_NC_001526.2__Human_papillomavirus_type_16' in the case of our simulated data, from the virus DB and store it as a file named 'results-virus-top1.fa' under the folder 'step2'. This file is used as a separate pseudo-chromosome 'chrVirus' to be concatenated with the reference human chromosome for the detection of virus integration sites in step (3).

Table 3: The top-ranking viruses identified from our simulation data

Virus name	Contig	Contig length (bp)	Mapped length /rate of contigs	# Reads fallen on contigs
gi_310698439_ref_NC_001526.2__Human_papillomavirus_type_16	comp4_c0_seq1	8253	7913/ 98.98	3916
gi_56463023_gb_AY686584.1__Human_papillomavirus_type_16_isolate_Qv17722E	comp4_c0_seq1	8253	7909/99.03	3916
gi_56462996_gb_AY686581.1__Human_papillomavirus_type_16_isolate_Qv15521E	comp4_c0_seq1	8253	7909/98.99	3916

Another file 'contig.txt' includes all high quality non-human contigs. Table 4 shows the first contig in the 'contig.txt' file created for our simulation data.

Table 4: The first contig in the file contig.txt created for our simulation data

Contig name	# Reads	Virus	E-value	Bit score	Sequence
comp4_c0_seq1	3916	gi_56463023_gb_AY686584.1__Human_papillomavirus_type_16_isolate_Qv17722E	0	1.42E+04	AGGTTCTAG CAATTGTCGT GCCTCAG……

4.3 Output of integration site detection

The third file, integration-sites.txt, reports the positions of all detected virus insertion sites. In our simulation data, we plugged one mutated copy of the reference HPV-16 virus at the position chr1:24020700 of the human genome. The following table provides the content of the integration-sites.txt file created by VirusFinder for this simulation data.

Table 5: The content of file integration-sites.txt created by VirusFinder for our simulation data

Chromosome 1	Position 1	Strand 1	Chromosome 2	Position 2	Strand 2	#Supporting reads (pair+softclipping)
chr1	24,020,709	+	chrVirus	1	+	13+12

The virus integration site in Table 5 involves two breakpoints, one at position 24,020,709 of the human chromosome 1 and another at position 1 of the HPV-16 virus sequence. The last column indicates how many NGS reads support this detection. The word *pair* means the number of paired-end reads with one end mapped to the human genome and another end aligned to the HPV-16 virus sequence. The word *softclipping* means the number of reads that actually harbor the integration breakpoint within themselves.

It may be worth mentioning that VirusFinder's predictions of virus integration sites are very close to the real positions. On most WGS samples, the virus integration loci predicted by VirusFinder are only several base pairs difference from the real ones. However, VirusFinder's ability to predict virus insertion is affected by sequencing coverage, read quality, read mapping, difference between the human genome and virus sequence under study, etc. This is the case not only to VirusFinder, but also true to other virus

insertion-detecting tools. Low quality of any of these factors could result in the deterioration in detection performance.

5. REFERENCE

Bhaduri, A., *et al*. (2012) Rapid identification of nonhuman sequences in high throughput sequencing data sets, *Bioinformatics*, **28**, 1174-1175.
